# Frequency of eating home cooked meals and potential benefits for diet and health: cross-sectional analysis of a population-based cohort study

**DOI:** 10.1186/s12966-017-0567-y

**Published:** 2017-08-17

**Authors:** Susanna Mills, Heather Brown, Wendy Wrieden, Martin White, Jean Adams

**Affiliations:** 1 Institute of Health & Society, Newcastle University, Baddiley-Clark Building, Richardson Road, Newcastle upon Tyne, NE2 4AX UK; 2 Human Nutrition Research Centre, Institute of Health & Society, Newcastle University, M1.151 William Leech Building, Medical School, Framlington Place, Newcastle upon Tyne, NE2 4HH UK; 3 Centre for Diet and Activity Research (CEDAR), MRC Epidemiology Unit, School of Clinical Medicine, University of Cambridge, Box 285 Biomedical Campus, Cambridge, CB2 0QQ UK

**Keywords:** Home cooking, Diet, Cardio-metabolic health

## Abstract

**Background:**

Reported associations between preparing and eating home cooked food, and both diet and health, are inconsistent. Most previous research has focused on preparing, rather than eating, home cooked food; used small, non-population based samples; and studied markers of nutrient intake, rather than overall diet quality or health. We aimed to assess whether frequency of consuming home cooked meals was cross-sectionally associated with diet quality and cardio-metabolic health.

**Methods:**

We used baseline data from a United Kingdom population-based cohort study of adults aged 29 to 64 years (*n* = 11,396). Participants self-reported frequency of consuming home cooked main meals. Diet quality was assessed using the Mediterranean Diet Score, Dietary Approaches to Stop Hypertension (DASH) score, fruit and vegetable intake calculated from a 130-item food frequency questionnaire, and plasma vitamin C. Markers of cardio-metabolic health were researcher-measured body mass index (BMI), percentage body fat, haemoglobin A_1c_ (HbA_1c_), cholesterol and hypertension. Differences across the three exposure categories were assessed using linear regression (diet variables) and logistic regression (health variables).

**Results:**

Eating home cooked meals more frequently was associated with greater adherence to DASH and Mediterranean diets, greater fruit and vegetable intakes and higher plasma vitamin C, in adjusted models. Those eating home cooked meals more than five times, compared with less than three times per week, consumed 62.3 g more fruit (99% CI 43.2 to 81.5) and 97.8 g more vegetables (99% CI 84.4 to 111.2) daily. More frequent consumption of home cooked meals was associated with greater likelihood of having normal range BMI and normal percentage body fat. Associations with HbA_1c_, cholesterol and hypertension were not significant in adjusted models. Those consuming home cooked meals more than five times, compared with less than three times per week, were 28% less likely to have overweight BMI (99% CI 8 to 43%), and 24% less likely to have excess percentage body fat (99% CI 5 to 40%).

**Conclusions:**

In a large population-based cohort study, eating home cooked meals more frequently was associated with better dietary quality and lower adiposity. Further prospective research is required to identify whether consumption of home cooked meals has causal effects on diet and health.

**Electronic supplementary material:**

The online version of this article (doi:10.1186/s12966-017-0567-y) contains supplementary material, which is available to authorized users.

## Background

The prevalence of obesity and diet-related non-communicable diseases (NCDs), such as type II diabetes, hypertension, and certain cancers, have been increasing steadily worldwide [1]. These changes have been accompanied by a decrease in the time spent cooking at home in the majority of developed countries [2, 3]. Concern has been expressed by policy makers, practitioners and researchers in the field of food and nutrition regarding a perceived decline in cooking skills, which has been hypothesised to be linked to the increase in diet-related NCDs [4–6].

Certain studies, primarily cross-sectional in design, have indicated that a higher frequency of cooking and preparing food at home may be associated with consuming a healthier diet [7–9] and benefits to health and longevity [10–12]. In contrast, other cross-sectional research has suggested that home food preparation and cooking may be associated with diets lower in fibre and higher in fat, saturated fat, sugar, and salt [13, 14] and could potentially be detrimental to health [15, 16]. Adding to this confusion, the majority of research to date has used cooking and food preparation practices as an exposure, rather than the consumption of home cooked food itself. Since eating food is more proximal to potential diet and health outcomes, focusing on behaviour upstream may be more likely to introduce confounding, for example regarding gender – given that more women than men engage in food preparation [3], and women tend to have healthier diets [17]. Of key primary interest therefore is establishing whether consuming home cooked meals is associated with benefits to diet and health, and subsequently investigating who eats home cooked meals, and then who prepares these meals and why.

To date, research investigating the potential advantages and disadvantages for diet and health of cooking and preparing food at home has generally focused on specific dietary indicators, rather than overall diet quality or health, and assessed measures cross-sectionally or after a brief follow-up period [18]. Most studies have been small in size, with associated limited scope to identify significant associations [8]; limited to a specific geographical area [19]; and/or restricted to population subgroups by for example age [10, 20] or ethnicity [11].

Despite the fact that the evidence base for relationships between cooking and both diet and NCDs is mixed and inconclusive, the promotion of home cooking forms part of public health strategies to improve diets and reduce obesity and diet-related NCDs internationally [21]. Further research is therefore crucial, to investigate on a large scale the potential associations between consumption of home cooked meals and diet and health outcomes.

In this study we aimed to assess whether the consumption frequency of home cooked meals was cross-sectionally associated with indicators of diet and cardio-metabolic status. In view of the current evidence base, we hypothesised that eating home cooked meals more frequently would be associated with markers of a healthier diet and improved cardio-metabolic health.

## Methods

### Data source

The Fenland Study is a population-based cohort study investigating interactions between genetic and lifestyle factors in determining obesity and diabetes. The study recruited adults born between 1950 and 1975 from general practice lists in Cambridgeshire, United Kingdom (UK), between 2005 and 2015 [22]. Participants were invited to attend one of three clinical sites in Cambridgeshire to take part in a detailed assessment. A total of 12,434 participants undertook baseline assessment (approximate response rate 27%), which involved a range of clinical, biological and anthropometric measurements, and completion of questionnaires. The data collection tools are available online [23].

Study exclusion criteria included previously diagnosed diabetes, psychosis, terminal illness, pregnancy, and inability to walk unaided. The Fenland study was approved by the Health Research Authority National Research Ethics Service Committee – East of England Cambridge Central – and performed in accordance with the Declaration of Helsinki. All participants provided written informed consent to participate in the study.

### Frequency of consumption of home cooked meals

Exposure was derived from an item in the participant questionnaire: ‘When eating your main meal at home, how often do you usually eat home cooked meals?’ Response categories were: never or rarely; one to two times per week; three to five times per week; or more than five times per week. The first two response categories were collapsed to yield appropriate numbers for statistical analysis, as previously [24], giving a three category variable: less than three times per week, three to five times per week, and more than five times per week.

### Indicators of diet quality

We assessed a range of dietary outcome variables, namely Mediterranean Diet Score (MDS) [25], Dietary Approaches to Stop Hypertension (DASH) score [26], plasma vitamin C, and fruit and vegetable intakes. Participants completed a 130-item, semi-quantitative food frequency questionnaire (FFQ) for their food intake over the previous year [27], which has been shown to yield valid and reproducible food intake assessments, and has been validated previously in dietary data collection in the European Prospective Investigation into Cancer and Nutrition (EPIC) studies [28]. The FFQ EPIC Tool for Analysis was used to convert food intake frequency to energy, nutrient and food intakes [29]. Total daily intake was provided in grams for carbohydrate, fibre, fat, saturated fat, sugar, protein, fruit, vegetables and alcohol. Total daily sodium intake was measured in milligrams, and total daily energy intake in kilojoules. Dietary intake values were winsorized at 1st and 99th percentiles, by replacing the smallest and largest percentage values in the distribution with the observations closest to them [30]. This was undertaken to account for their positively skewed distribution, and the limitations of the FFQ as a tool to collect precise data on dietary intake [31, 32]. Data on dietary supplements were not collected.

The consumption of a more DASH accordant diet is associated with positive health indicators and lowered cardio-metabolic risk [33–35]. The DASH diet assumes that beneficial impact is derived from the overall diet, rather than individual foods or nutrients playing important roles [36]. A DASH score was computed from each participant’s dietary intake using the method developed by Fung et al. [26]. This index includes eight components (one nutrient and seven food groups) based on eating guidance from the United States (US) National Heart, Lung and Blood Institute [37]. Scoring is established through quintile rankings, on the basis of relative comparisons to the rest of the sample, with men and women classified separately. Participants are allocated a score from one (lowest quintile) to five (highest quintile) for energy-adjusted intake of: low-fat dairy products; whole grains; nuts, seeds and legumes; fruit (includes fruit juice); and vegetables (excludes potatoes). In contrast, for intakes of red and processed meat; sodium; and sugar-sweetened beverages, participants are allocated a score from one (highest quintile) to five (lowest quintile). Scores are then combined to give a total DASH score, ranging from a minimum of eight to a maximum of 40 points. In this study, DASH scores were standardised using the z-score, to yield a semi-continuous measure of participants’ relative standing.

The Mediterranean diet is generally considered to be low in consumption of red meats, moderate in consumption of fish, poultry, fermented dairy products and wine, and high in consumption of fruits, legumes, cereals and olive oil [38, 39]. Concordance with the Mediterranean diet has been linked with positive health outcomes, in particular the primary prevention of cardiovascular disease [40]. A Mediterranean diet score (MDS) was calculated from each participant’s dietary intake using sex-specific tertiles, according to relative comparisons with the rest of the sample. Scores of zero, one or two were allocated for each of nine dietary components, including legumes; fruit and nuts; vegetables; ratio of monounsaturated and polyunsaturated fatty acids to saturated fatty acids; fish; meat products; dairy products; cereals; and alcohol [25]. In order to appraise quality of diet independent of quantity, dietary intakes were adjusted to a 2000 kcal/day diet using the residual method. This also aimed to help reduce measurement errors, since energy intake is partially associated with over-reporting and under-reporting of dietary intake [41]. MDS scores were then standardised using the z-score.

Plasma vitamin C (μmol/l) provides an objective biomarker of fruit and vegetable consumption [42] and fruit and vegetable intake is promoted in dietary guidelines [43, 44]. Fasting venous blood samples drawn into heparin-containing tubes and stabilised using metaphosphoric acid (10%) were measured for plasma vitamin C levels by fluorometric assay within two months, as undertaken previously [45].

### Markers of cardio-metabolic health

We used body mass index (BMI), percentage body fat, haemoglobin A_1c_ (HbA_1c_), cholesterol and hypertension as indicators of cardio-metabolic health. Elevated total cholesterol and low levels of high density lipoprotein cholesterol (HDL) are associated with increased risk of cardiovascular disease [46], and the derived ratio of total cholesterol to HDL is used in the QRISK2 model to estimate risk of cardiovascular disease over the next ten years [47]. HDL and total cholesterol were measured in mmol/l in fasting venous blood samples, and the ratio of total cholesterol to HDL calculated for analysis. In line with UK guidance, a ratio of 4.0 or greater was used to indicate higher risks to cardio-metabolic health [48].

Excess body fat and raised BMI have been associated with increased risk of various NCDs [49]. Height and weight were measured at the clinical sites by trained observers, with participants wearing light clothing and barefoot. Height was measured to the nearest 0.1 cm using a wall-mounted calibrated stadiometer (SECA 240, Birmingham, UK). Weight was measured to the nearest 0.1 kg with a calibrated electronic scale (TANITA, BC-418MA, Tokyo, Japan). BMI was derived as weight (kg) divided by height (m^2^). Dual-energy X-ray absorptiometry (DEXA; Lunar prodigy advanced fan beam scanner (GE Healthcare)) was used to assess body composition, and has been described in detail elsewhere [22]. A three-compartment model (fat mass, fat-free mass and bone mineral mass) was used to estimate percentage total body fat. In line with international guidance, overweight was defined as BMI 25 kg/m^2^ and above [50] and excess percentage body fat as 25% and over for males and 38% and over for females [51].

Haemoglobin A_1c_ (HbA_1c_) has previously been used to assess risk of developing type II diabetes [52]. Participants’ HbA_1c_ was measured on entry to the study from fasting venous blood samples, in either mmol/mol or as a percentage. A conversion algorithm was used to convert all measurements to mmol/mol, and in accordance with international guidance [52], a level of 42.00 mmol/mol (6.0%) or higher was used to indicate increased risk of type II diabetes.

Hypertension is associated with an elevated risk of developing cardiovascular disease [53]. Using an upper arm cuff and automated oscillometric device, three sets of diastolic and systolic blood pressure measurements were performed on each participant. The first readings were discarded and the lowest systolic and lowest diastolic readings from the last two readings were used for assessment. In adherence to UK guidance [54], readings of at least 90 mmHg diastolic and 140 mmHg systolic were considered indicative of hypertension. Participants currently taking hypotensive medication, or self-reporting a diagnosis of hypertension from a clinician, were also classified as hypertensive.

### Covariates

In view of the current evidence base regarding factors influencing dietary intake [55], a self-administered questionnaire was used to collect demographic and behavioural variables including sex, age, smoking status (current/ex-smoker or never smoker), and first degree family history of relevant diseases such as type II diabetes. Participants were asked whether or not they had been employed in the past four weeks, and those answering yes were identified as currently working. Participants reporting more than 48 h working in any one week were identified as working overtime. Socioeconomic status was assessed using age at leaving full time education, which was divided into three categories: education up to age 16 years (compulsory education); over 16 and up to 18 years (post-compulsory school education); and over 18 years (higher education).

Physical activity was measured objectively using an integrated movement and heart rate sensor (Actiheart; CamNtech, Cambridge, UK) attached to the chest via two standard ECG electrodes and worn during free-living over six days [56]. A ramped treadmill protocol test was used to individually calibrate heart rate, as undertaken previously [57]. Monitoring data were cleaned for measurement issues and sensor wear time was specified as at least 48 h, although data were not necessarily spread over a full 24 period. Periods of non-wear were inferred from the combination of non-physiological heart rate and prolonged periods of inactivity, which were taken into account to minimise diurnal information bias when summarising the intensity time-series. Data were processed [58] and a branched equation framework [59] used for modelling to estimate intensity time series. These were collated over time to yield daily physical activity energy expenditure (kJ/kg per day).

### Statistical analysis

All analyses were on a complete case basis. Thus, participants with missing data on any of the variables described were excluded (*n* = 1038), leaving 11,396 participants (91.7% total cohort) in the analysis. The outcome variable with the greatest missingness was vitamin C (missing for 350 participants) and the covariate with the greatest missingness was physical activity (missing for 227 participants). Differences in the characteristics of Fenland study participants included and excluded from the analytic sample were tested using the Mann–Whitney test for continuous variables and Pearson Chi squared test for categorical variables.

Differences in covariates and markers of diet and cardio-metabolic health across the three frequency categories of consuming home cooked meals were assessed using descriptive statistics (Kruskal-Wallis test and Pearson Chi squared test). Separate analyses were then run for each outcome variable, using linear regression for continuous diet variables and logistic regression for binary health variables. Analyses were adjusted for covariates: sex, age, alcohol intake, smoking status, age at leaving full-time education, physical activity, working status, and overtime working, with supplementary adjustment for family history of diabetes for the outcome of HbA_1c_. The analyses for markers of cardio-metabolic status were additionally adjusted for dietary variables (MDS, DASH score, plasma vitamin C, fruit and vegetable intakes) to assess the potential health benefits of consuming home cooked meals independent of dietary improvements.

All analyses were conducted using Stata (version 14; Stata Corp.) and in view of the large number of comparisons, 99% confidence intervals were used to determine if variables were statistically significant (see Additional file 1 for details of the participant sample).

## Results

Participant distribution is summarised in Additional file 2. A slight majority of the included sample was female (53.3%), with median age 48.9 years. Most participants were non-smoking (88.2%), with no family history of diabetes (76.1%), median alcohol intake of 5.47 g/day and physical activity expenditure of 51.0 kJ/kg/day. Most participants had left full time education by 18 years of age (62.2%), were currently in work (82.8%), and did not work overtime (88.8%). There were significant differences between the included and excluded participants in terms of sex, age, smoking status, physical activity expenditure, working status, and frequency of consuming home cooked meals.

Table 1 shows that 6.2% of included participants consumed home cooked meals as their main meal less than three times per week, 32.4% consumed these three to five times per week, and 61.5% consumed these more than five times per week. Participants who ate home cooked meals more frequently tended to be female, older, non-smokers, not currently in work, working fewer hours and not working overtime, older at leaving full time education, with greater daily alcohol intake. These associations were all statistically significant at *p* < 0.01. Participants who consumed home cooked meals more frequently generally had higher plasma vitamin C, higher fruit and vegetable intakes, and higher MDS and DASH score. They were also less likely to have an overweight BMI, excess percentage body fat, high risk cholesterol ratio, or to be at risk of developing diabetes according to HbA_1c_ level.

**Table 1:** Characteristics of participants overall and by frequency of consuming home cooked meals

Covariate^a^		Consumption of home cooked main meals			
		Total	<3×/week	3-5×/week	>5×/week
		*n* = 11,396 (100.00%)	*n* = 704 (6.18%)	*n* = 3688 (32.36%)	*n* = 7004 (61.46%)
Sex	Male	5321	389 (7.31)	1914 (35.97)	3018 (56.72)
	Female	6075	315 (5.19)	1774 (29.20)	3986 (65.61)
Age (years)	Median (IQR)	48.9 (42.7, 54.8)	47.1 (41.7, 53.3)	48.3 (42.2, 53.9)	49.5 (43.1, 55.3)
Alcohol (grams/day)	Median (IQR)	5.47 (1.27, 10.72)	3.90 (0.76, 9.56)	5.47 (1.30, 10.56)	5.47 (1.27, 10.88)
Age at leaving full-time education (years)	≤16	4570	351 (7.68)	1709 (37.40)	2510 (54.92)
	>16 to ≤18	2521	148 (5.87)	839 (33.28)	1534 (60.85)
	>18	4305	205 (4.76)	1140 (26.48)	2960 (68.76)
Smoker	No	10,045	569 (5.66)	3133 (31.19)	6343 (63.15)
	Yes	1351	135 (9.99)	555 (41.08)	661 (48.93)
Family History of diabetes^b^	No	8677	535 (6.17)	2796 (32.22)	5346 (61.61)
	Yes	2719	169 (6.22)	892 (32.81)	1658 (60.98)
Physical activity (kJ^c^/kg^d^/day)	Median (IQR)	51.00 (37.84, 66.75)	49.64 (35.82, 65.82)	51.57 (38.22, 67.64)	50.89 (37.88, 66.27)
Working in past 4 weeks	No	1959	118 (6.02)	563 (28.74)	1278 (65.24)
	Yes	9437	586 (6.21)	3125 (33.11)	5726 (60.68)
Working hours	Median (IQR)	33.0 (14.0, 40.0)	37.0 (20.0, 43.7)	35.0 (17.5, 41.0)	30.0 (12.0, 40.0)
Overtime work (>48 h/week)	No	10,116	592 (5.85)	3243 (32.06)	6281 (62.09)
	Yes	1280	112 (8.75)	445 (34.77)	723 (56.48)
Outcome^i^					
Vitamin C (umol/l^e^)	Median (IQR)	69.40 (56.00, 82.00)	63.15 (44.73, 77.38)	66.80 (52.70, 80.10)	71.1 (58.5, 83.4)
Fruit intake (grams/day)	Median (IQR)	207.10 (111.61, 329.50)	142.53 (60.08, 264.19)	180.53 (93.10, 293.10)	226.83 (131.16, 353.04)
Vegetable intake (grams/day)	Median (IQR)	258.95 (188.89, 348.56)	174.41 (111.92, 257.26)	234.59 (172.55, 310.33)	280.56 (209.53, 375.83)
DASH score^f^	Median (IQR)	24 (21, 27)	22 (19, 25)	23 (20, 26)	25 (22, 28)
MDS^g^	Median (IQR)	9 (7,11)	7 (6, 10)	8 (6, 10)	10 (7, 11)
Excess body fat (≥25% men; ≥38% women)	No	4831	246 (5.09)	1399 (28.96)	3186 (65.95)
	Yes	6565	458 (6.98)	2289 (34.87)	3818 (58.16)
Overweight BMI^h^ (≥25.0)	No	4384	211 (4.81)	1290 (29.43)	2883 (65.76)
	Yes	7012	493 (7.03)	2398 (34.20)	4121 (58.77)
High cholesterol ratio (≥4.0)	No	7234	400 (5.53)	2209 (30.54)	4625 (63.93)
	Yes	4162	304 (7.30)	1479 (35.54)	2379 (57.16)
High HbA_1c_^i^(≥42.00)	No	10,207	608 (5.96)	3265 (31.99)	6334 (62.06)
	Yes	1189	96 (8.07)	423 (35.58)	670 (56.35)
Hypertension	No	8561	516 (6.03)	2761 (32.25)	5283 (61.72)
	Yes	2836	188 (6.63)	927 (32.69)	1721 (60.68)

^a^Results shown as number (row percentage). Median (inter-quartile range) shown for: age, alcohol, physical activity, average working hours, vitamin C, fruit intake, vegetable intake, DASH score, MDS

^b^History of diabetes in first degree relative

^c^kj = kilojoules

^d^kg = kilograms

^e^umol/l = micromole/l

^f^DASH = Dietary Approaches to Stop Hypertension

^g^MDS = Mediterranean Diet Score

^h^BMI = body mass index

^i^HbA_1c_ = Haemoglobin A_1c_

Multivariate associations between the frequency of consuming home cooked meals and indicators of diet quality and cardio-metabolic status are shown in Table 2. In all cases, consuming home cooked meals more frequently was significantly associated with indicators of a healthier diet, as measured by higher MDS, DASH score, plasma vitamin C, and fruit and vegetable intakes. These associations were present for both consuming home cooked meals three to five times per week, and more than five times per week, compared with the reference of less than three times per week, and remained robust to adjustment for sociodemographic and behavioural covariates. In particular, those who consumed home cooked meals more than five times per week consumed 62.3 g more fruit (99% CI 43.2 to 81.5) and 97.8 g more vegetables (99% CI 84.4 to 111.2) daily than those who consumed home cooked meals less than three times per week. This equates to more than three-quarters of a portion of fruit, and almost one and a quarter portions of vegetables, or approximately two extra portions of fruit and vegetables per day.

**Table 2:** Associations between frequency of consuming home cooked meals and markers of diet and cardio-metabolic status

Outcomes	Home cooked meals^a^	Unadjusted value (99% CI^b^)	Adjusted value, model 1^c^ (99% CI^b^)	Adjusted value, model 2^d^ (99% CI^b^)
Regression coefficients for dietary indicators				
DASH score^e^	3-5×/week	0.23 (0.13, 0.34)	0.18 (0.08, 0.28)	NA
	>5×/week	0.61 (0.51, 0.71)	0.44 (0.35, 0.54)	NA
MDS^f^	3-5×/week	0.27 (0.17, 0.38)	0.23 (0.13, 0.33)	NA
	>5×/week	0.64 (0.54, 0.74)	0.52 (0.42, 0.61)	NA
Vitamin C (μmol/l^g^)	3-5×/week	4.50 (2.28, 6.73)	3.29 (1.18, 5.39)	NA
	>5×/week	8.95 (6.81, 11.09)	5.35 (3.31, 7.39)	NA
Fruit intake (grams/day)	3-5×/week	32.29 (12.14, 52.44)	27.17 (7.43, 46.92)	NA
	>5×/week	79.06 (59.69, 98.43)	62.33 (43.19, 81.46)	NA
Vegetable intake (grams/day)	3-5×/week	54.22 (40.06, 68.39)	50.54 (36.61, 64.29)	NA
	>5×/week	107.43 (93.81, 121.05)	97.83 (84.42, 111.24)	NA
Odds ratios for markers of cardio-metabolic status				
Cholesterol binary (high vs low risk)	3-5×/week	0.88 (0.71, 1.09)	0.96 (0.76, 1.21)	0.99 (0.78, 1.25)
	>5×/week	0.68 (0.55, 0.83)	0.87 (0.70, 1.09)	0.93 (0.74, 1.17)
BMI^h^ binary (over- vs normal weight)	3-5×/week	0.80 (0.63, 1.00)	0.81 (0.64, 1.02)	0.82 (0.65, 1.05)
	>5×/week	0.61 (0.49, 0.76)	0.70 (0.55, 0.88)	0.72 (0.57, 0.92)
Body fat binary (excess vs normal)	3-5×/week	0.88 (0.70, 1.10)	0.89 (0.71, 1.13)	0.92 (0.73, 1.17)
	>5×/week	0.64 (0.52, 0.80)	0.71 (0.57, 0.89)	0.76 (0.60, 0.95)
HbA_1c_^i^ binary (high vs low risk)	3-5×/week	0.82 (0.60, 1.12)	0.83 (0.60, 1.15)	0.86 (0.62, 1.19)
	>5×/week	0.67 (0.50, 0.91)	0.68 (0.49, 0.93)	0.73 (0.53, 1.01)
Hypertension binary (yes vs no)	3-5×/week	0.92 (0.72, 1.17)	0.88 (0.69, 1.13)	0.89 (0.69, 1.14)
	>5×/week	0.89 (0.71, 1.13)	0.84 (0.67, 1.07)	0.86 (0.67, 1.09)

^a^Consumption of home cooked meals as main meal at home: comparisons with low consumption (<3×/week, reference), for medium consumption (3-5×/week), and high consumption (>5×/week)

^b^CI = 99% confidence interval

^c^Adjusted for age, sex, alcohol intake, smoking, physical activity, working status, working overtime, years of full time education (+ family history diabetes for HbA_1c_ outcome)

^d^Adjusted for age, sex, alcohol intake, smoking, physical activity, working status, working overtime, years of full time education, DASH score, MDS, vitamin C, fruit intake, vegetable intake (+ family history diabetes for HbA_1c_ outcome)

^e^DASH = Dietary Approaches to Stop Hypertension

^f^MDS = Mediterranean Diet Score

^g^umol/l = micromole/l

^h^BMI = Body Mass Index

^i^HbA_1c_ = Haemoglobin A_1c_

In terms of cardio-metabolic status, consuming home cooked meals more than five times per week compared with the reference of less than three times per week was significantly associated with all markers except hypertension in the unadjusted models. After adjustment for sociodemographic and behavioural covariates (model 1), the association between consuming home cooked meals more than five times per week and high risk cholesterol ratio was extinguished. After further adjustment for dietary variables (model 2), only the associations with having a normal range BMI and lower percentage body fat remained significant. Such associations indicated that consuming home cooked meals more than five times per week compared with the reference was associated with improved adiposity, independent of the effects due to diet. Those consuming home cooked meals more than five times per week were 28% less likely to have a BMI in the overweight range (99% CI 8 to 43%), and 24% less likely to have excess percentage body fat (99% CI 5 to 40%), compared with those who consumed home cooked meals less than three times per week. Overall, a higher frequency of consuming home cooked meals was associated with markers of improved cardio-metabolic health, including lower risk cholesterol ratio, normal range BMI, lower percentage body fat, and lower risk of diabetes according to HbA_1c_ level.

## Discussion

### Statement of principal findings

In accordance with our hypothesis, a higher frequency of consuming home cooked main meals was significantly associated with indicators of a healthier diet, namely DASH score, MDS, plasma vitamin C, fruit intake and vegetable intake. Similarly, eating home cooked meals more frequently was significantly associated with several markers of cardio-metabolic health, including lower likelihood of having an overweight BMI, and lower likelihood of excess percentage body fat. Associations between frequency of home cooked meal consumption and markers of cardio-metabolic health were strongest at the highest consumption frequency of eating meals more than five times per week.

To our knowledge, this is the first large scale, population-based study to address associations between the frequency of consuming home cooked meals and indicators of both diet quality and cardio-metabolic status. The study has been reported according to the STROBE-nut guidelines [60] (see Additional file 3).

### Strengths and weaknesses of the study

The Fenland study is a large cohort, with detailed sociodemographic data, objective physical measurements and samples, and comprehensive dietary measures. Participants in this study were from the county of Cambridgeshire, which is representative of the wider population in England in terms of adult obesity and several lifestyle variables, such as smoking and levels of physical activity [61].

Overall diet quality was assessed using two composite diet scores, DASH and MDS. Using two composite scores provided robust evidence in support of potential associations between consuming home cooked meals more frequently and higher diet quality. These results were supported by similar associations with higher fruit and vegetable intakes, measured by both FFQ, and plasma vitamin C as a biomarker. We used consumption, rather than preparation, of home cooked meals as our exposure, which is likely to be closer on the potential causal pathway to diet and health outcomes. The use of objective measurements for determining cholesterol ratio, BMI, percentage body fat, HbA_1c_ level and hypertension is likely to increase the validity of these markers of cardio-metabolic status, and the confidence in conclusions drawn from resultant analyses.

This research is also subject to some limitations. The cross-sectional nature of the data means that direction of cause and effect cannot be established, although follow-up data collection in the Fenland study is currently underway, which will enable future longitudinal analysis. Participants were recruited between the ages of 29 and 64 years, and are therefore not representative of the full UK population age range. Given that food preparation practices vary with age [62], our results may not be generalizable to younger populations. We excluded participants with missing data on any of the analytic variables, and excluded participants were systematically different from the rest of the cohort in terms of certain characteristics (see Additional file 2). Furthermore, differences in cooking and food culture internationally may mean that the relationships between consuming home cooked meals, diet quality, and cardio-metabolic health, vary between countries. Therefore, our findings may not necessarily be generalizable to other populations.

The fruit and vegetable intakes and DASH and MDS dietary scores were derived from FFQ data, which although validated, may be subject to error and biases [31, 32]. The composite scores assessed diet quality relative to other participants, rather than establishing absolute values, and ranking groups may constitute a broad range. The exposure variable for consumption of home cooked meals was derived from a questionnaire item, and given the absence of consensus on home cooking terminology [63, 64], participants may have interpreted this question differently. We collected data specifically on home cooked meals eaten at home and not those eaten elsewhere, such as packed lunches taken to work or place of study. The self-reported nature of several sociodemographic and behavioural variables, such as smoking, may have led to variables being correlated with each other, with associated risk of type II analytical errors.

Although we adjusted for a number of relevant potential confounders in our analyses, residual confounding remains possible. If people who consume home cooked meals more frequently are also more likely to engage in other health promoting behaviours, this could artificially strengthen associations between increased consumption of home cooked meals and markers of cardio-metabolic health.

### Interpretation of findings in the context of existing research

Our findings reflect those of others that found associations between home food preparation and cooking and higher quality diets. A recent systematic review [18] identified that potential benefits included intake from healthier food groups [19, 65, 66]; greater fruit and vegetable preference and healthy eating self-efficacy [67]; enhanced nutrient intake [7, 68]; higher Diet Quality Index-International score and intake from healthier food groups [20]; trend towards higher Healthy Eating Index score [69]; consumption of a healthful dietary pattern [70]; and improved adherence to: Healthy People 2010 dietary intake objectives [8], Balance of Good Health (now Eatwell Guide) criteria [71], and a Mediterranean diet using the KIDMED index [72]. A greater frequency of home cooked meals has also been associated with higher Healthy Eating Index scores [24]. However, the majority of this research has been cross-sectional and therefore unable to conclusively indicate direction of causation. Most studies have also employed self-reported measures, which are vulnerable to bias [73], and have used food preparation practices as an exposure, rather than the consumption of home cooked food itself.

Our results also support previous studies that identified associations between home food preparation and cooking and potential advantages to health. Greater home cooking frequency has been linked with longer lifespan [10] and more frequent consumption of meals prepared at home has been associated with reduced risk of developing type II diabetes [12]. Amongst adolescents, healthier home cooking by a caregiver was linked with lowered risk of having an overweight or obese BMI [11]. However, our findings conflict with a US study that reported more time spent on home food preparation and associated clean-up at baseline, or increased involvement over time, was linked with an adverse cardio-metabolic profile [16]. Possible reasons for this discrepancy include that the US study used time spent preparing meals, rather than meal consumption, as the exposure, and the exposure included clean-up time, which may have a differential impact on cardio-metabolic health. Since food preparation activities are strongly patterned by gender [18, 74], this may also confound observed associations with health.

### Meaning of the study: possible mechanisms and implications for clinicians and policymakers

Our findings indicate that an increased frequency of consuming home cooked meals is associated cross-sectionally with markers of a healthier diet, and indicators of improved cardio-metabolic health, particularly in terms of adiposity, cholesterol and diabetes risk. Links between more frequent consumption of home cooked meals and dietary benefits could be attributable to healthier food preparation methods, increased dietary variety and/or consumption of healthier food groups. Such links may also be due to decreased intake of convenience foods, which tend to prioritise ingredients such as fat, sugar and salt to increase palatability and preservation, over those for optimising health [75].

The association between a higher frequency of consuming home cooked meals and potential benefits for health in terms of hypertension was not significant in the unadjusted model, and in terms of cholesterol was no longer significant after adjustment for sociodemographic and behavioural variables. This may be because the hypertension variable was poorly ascertained, since in addition to blood pressure measurement, participants were required to report on any previous diagnoses of hypertension, and receipt of hypotensive medication. However, we conducted a sensitivity analysis for the relationship between frequency of consuming home cooked meals and hypertension, with the inclusion and the exclusion of participants diagnosed with hypertension by a doctor and/or receiving hypotensive medication. Regardless of whether or not these participants were excluded, the relationship was not significant. Cholesterol is strongly genetically determined [76], and the impact of home cooked meal consumption may not have been sufficient to result in statistically significant changes.

The cross-sectional association between higher frequency of consuming home cooked meals and lower adiposity was robust to adjustment for sociodemographic, lifestyle, and dietary covariates, whilst the association with lower likelihood of being classified as at risk of diabetes according to HbA_1c_ level was borderline significant. Although the direction of causation cannot be established, this indicates that home cooking potentially confers benefits to health, beyond those mediated through dietary changes. Such benefits from eating home cooked meals might be attributable to consumption of smaller portion sizes [77]; moderated snacking behaviour [78]; more structured mealtimes and the time of day at which meals are consumed [79]. Increased social cohesion has been linked with potential health benefits [80], and it is plausible that higher social capital may be associated with more sociable eating patterns. Given the potential time and effort involved in home cooking, home cooked meals may be more likely to be shared together than meals from other sources, and a range of benefits to diet, health and wellbeing derived from shared mealtimes have been identified [81, 82].

Our results support previous research indicating putative benefits from home cooked meals, suggesting that public health promotional messages should advocate for cooking at home as a positive approach for improving diet and health. Strategies could also be considered for supporting people to learn to cook healthy meals, and to use their skills often, for example using digital technology and social media to provide shopping list generators, food preparation teaching videos, and nutritional information. Regularity is particularly important, given that our findings indicated the greatest potential advantages from consuming home cooked meals were experienced at the highest frequencies of consumption. Infrequent home cooking, such as a weekly Sunday lunch, is unlikely to be of benefit to population health, and cooking habits should be adopted as part of the daily routine. This is in accordance with research suggesting that routinized home cooking behaviour is more likely to be maintained and prioritised over time [83].

### Unanswered questions and future research

The evidence base for associations between home cooking, dietary indicators and cardio-metabolic status requires further longitudinal studies to establish causal relationships. This could be facilitated by incorporating questions on home cooking into current large scale national longitudinal surveys, particularly those with more detailed existing dietary components. Additional analyses, for example using structural equation modelling, could be employed to explore causal pathways more fully in future. It will also be insightful to identify who eats home cooked meals and why, and then who prepares these meals and why. Other questions include exploring further the potential benefits of home cooking beyond those mediated through diet, and determining the most effective approaches to encourage home cooking, which may require a combination of tailored interventions.

## Conclusion

In a cross-sectional population-based study, consuming home cooked main meals more frequently was associated with a range of indicators of a healthier diet, and several markers of cardio-metabolic health including adiposity, cholesterol and diabetes risk. Strongest associations were observed for the highest frequency of consuming home cooked meals, more than five times per week. These findings suggest that regularly eating home cooked meals may confer benefits to diet and health, and that home cooking promotion and skill development should form part of future public health initiatives. Further research regarding causal relationships between home cooking, diet and health; the wider social aspects of home food preparation; and evaluation of interventions to promote home cooking, is required.

## Additional files

Additional file 1: This file provides requested information regarding how the sample was recruited, how representative the sample was of the target group, how the analysed sample differed from the recruited sample, and how missing data were handled. (DOCX 17 kb)

Additional file 2: Characteristics of Fenland study participants included and excluded from the analytic sample. This table compares the characteristics of participants in the Fenland study who were included in the current study analytic sample, and those who were excluded. (DOCX 24 kb)

Additional file 3: STROBE-nut: An extension of the STROBE statement for nutritional epidemiology. This table provides a checklist, reporting adherence of the current study to the STROBE-nut guidelines. (DOCX 34 kb)

## Abbreviations

BMI: Body mass index
DASH: Dietary Approaches to Stop Hypertension
EPIC: European Prospective Investigation into Cancer and Nutrition
FFQ: Food Frequency Questionnaire
HbA_1c_: Haemoglobin A_1c_
HDL: High density lipoprotein
MDS: Mediterranean Diet Score
NCDs: Non-communicable diseases
UK: United Kingdom
US: United States
