# Supplementary material for: Frequency of eating home cooked meals and potential benefits for diet and health: cross-sectional analysis of a population-based cohort study
Source: Int J Behav Nutr Phys Act. 2017 Aug 17;14:109. doi: 10.1186/s12966-017-0567-y (PMC5561571; doi:10.1186/s12966-017-0567-y)
Supplement: Supplementary file 1 — This file provides requested information regarding how the sample was recruited, how representative the sample was of the target group, how the analysed sample differed from the recruited sample, and how missing data were handled. (DOCX 17 kb) [file 12966_2017_567_MOESM1_ESM.docx]

**Additional file 1: Details of participant sample for editors and reviewers**

1. **How the sample was recruited**

Details of how the sample was recruited are provided in the methods section:

Data source

The Fenland Study is a population-based cohort study investigating interactions between genetic and lifestyle factors in determining obesity and diabetes. The study recruited adults born between 1950 and 1975 from general practice lists in Cambridgeshire, United Kingdom (UK), between 2005 and 2015 [1]. Participants were invited to attend one of three clinical sites in Cambridgeshire to take part in a detailed assessment. A total of 12,434 participants undertook baseline assessment, which involved a range of clinical, biological and anthropometric measurements, and completion of questionnaires. The data collection tools are available online [2].

Study exclusion criteria included previously diagnosed diabetes, psychosis, terminal illness, pregnancy, and inability to walk unaided. The Fenland study was approved by the Health Research Authority National Research Ethics Service Committee – East of England Cambridge Central – and performed in accordance with the Declaration of Helsinki. All participants provided written informed consent to participate in the study.

1. **How representative the sample was of the target group**

Fenland study participants were recruited between the ages of 29 and 64 years, and therefore do not represent the full age range of the UK population. People with previously diagnosed diabetes, psychosis, terminal illness, pregnancy, and inability to walk unaided were excluded. Participants were recruited from general practice lists, and therefore those not registered with a practice were not eligible for recruitment. The Fenland study sample was otherwise representative of the population of Cambridgeshire, UK.

We did not have access to data on the response rate of potential study participants to invitations to take part in the Fenland study.

1. **How the analysed sample differed from the recruited sample**

The analytic sample (n=11,396) differed in some respects from the recruited Fenland study sample (n=12,434). These differences are shown in the table in Additional file 2.

1. **How any missing data were handled**

All analyses were on a complete case basis. Thus, participants with missing data on any of the variables described were excluded (n=1,038), leaving 11,396 participants (91.7% total cohort) in the analysis.

**References**

1. de Lucia Rolfe E, Loos RJF, Druet C, Stolk RP, Ekelund U, Griffin SJ, et al. Association between birth weight and visceral fat in adults. Am J Clin Nutr. 2010;92:347-352.

2. MRC Epidemiology Unit. Fenland Study. 2014. <http://epi-meta.medschl.cam.ac.uk/includes/fenland/fenland.html>. Accessed 04 May 2017.
