# Supplementary material for: Frequency of eating home cooked meals and potential benefits for diet and health: cross-sectional analysis of a population-based cohort study
Source: Int J Behav Nutr Phys Act. 2017 Aug 17;14:109. doi: 10.1186/s12966-017-0567-y (PMC5561571; doi:10.1186/s12966-017-0567-y)
Supplement: Supplementary file 2 — Characteristics of Fenland study participants included and excluded from the analytic sample. This table compares the characteristics of participants in the Fenland study who were included in the current study analytic sample, and those who were excluded. (DOCX 24 kb) [file 12966_2017_567_MOESM2_ESM.docx]

**Additional file 2:** Characteristics of Fenland study participants included and excluded from the analytic sample

| Variable^[[1]](#footnote-1)^ | | Included  n = 11,396 (91.65%) | Excluded  n = 1,038 (8.35%) | Statistical tests^[[2]](#footnote-2)^ |
| --- | --- | --- | --- | --- |
| Sex | **Male** | 5,321 (46.69) | 422 (40.66) | χ^2^(1) = 13.95, p<0.0001 |
|  | **Female** | 6,075 (53.31) | 616 (59.34) |  |
| Age (years) | **Median (IQR)** | 48.9 (42.7, 54.8) | 48.0 (42.2, 53.5) | z = -3.69, p=0.0002 |
| Alcohol (grams/day) | **Median (IQR)** | 5.47 (1.27, 10.72) | 5.14 (0.79, 10.88) | z = -0.94, p=0.35 |
| Age at leaving full-time education (years) | **≤16** | 4,570 (40.10) | 362 (38.39) | χ^2^(2) = 1.85, p=0.40 |
|  | **>16 to ≤18** | 2,521 (22.12) | 204 (21.63) |  |
|  | **>18** | 4,305 (37.78) | 377 (39.98) |  |
| Smoker | **No** | 10,045 (88.14) | 742 (83.75) | χ^2^(1) = 14.87, p<0.0001 |
|  | **Yes** | 1,351 (11.86) | 144 (16.25) |  |
| Family history of diabetes^[[3]](#footnote-3)^ | **No** | 8,677 (76.14) | 798 (76.88) | χ^2^(1) = 0.29, p=0.59 |
|  | **Yes** | 2,719 (23.86) | 240 (23.12) |  |
| Physical activity (kJ^[[4]](#footnote-4)^/kg^[[5]](#footnote-5)^/day) | **Median (IQR)** | 51.00 (37.84, 66.75) | 48.25 (34.45, 64.61) | z = -3.70, p=0.0002 |
| Working in past 4 weeks | **No** | 1,959 (17.19) | 217 (20.91) | χ^2^(1) = 9.10, p=0.003 |
|  | **Yes** | 9,437 (82.81) | 821 (79.09) |  |
| Overtime work (>48 hours/ week) | **No** | 10,116 (88.77) | 896 (89.60) | χ^2^(1) = 0.64, p=0.42 |
|  | **Yes** | 1,280 (11.23) | 104 (10.40) |  |
| Home cooked meal consumption | **<3x/week** | 704 (6.18) | 79 (7.79) | χ^2^(2) = 10.10, p=0.006 |
|  | **3-5x/week** | 3,688 (32.36) | 360 (35.50) |  |
|  | **>5x/week** | 7,004 (61.46) | 575 (56.71) |  |

1. Results shown as number (column percentage). Median (inter-quartile range) shown for: age, alcohol, physical activity [↑](#footnote-ref-1)
2. Testing for significant differences between included and excluded populations using Mann-Whitney test with z-scores for continuous variables, and Pearson Chi squared test for categorical variables. Significance at 1% level [↑](#footnote-ref-2)
3. History of diabetes in first degree relative [↑](#footnote-ref-3)
4. kj = kilojoules [↑](#footnote-ref-4)
5. kg = kilograms [↑](#footnote-ref-5)
